# Antibiofilm Activity of β-Lactam/β-Lactamase Inhibitor Combination against Multidrug-Resistant *Salmonella* Typhimurium

**DOI:** 10.3390/pathogens11030349

**Published:** 2022-03-13

**Authors:** Nana Nguefang Laure, Juhee Ahn

**Affiliations:** 1 Department of Biomedical Science, Kangwon National University, Chuncheon 24341, Korea; 202016039@kangwon.ac.kr; 2 Institute of Bioscience and Biotechnology, Kangwon National University, Chuncheon 24341, Korea

**Keywords:** ampicillin, ceftriaxone, sulbactam, antibiotic resistance, β-lactamase activity, anti-biofilm, *Salmonella*

## Abstract

This study was designed to assess the effect of β-lactam/β-lactamase inhibitor combinations on the inhibition of biofilm formation of *Salmonella* Typhimurium. The anti-planktonic and anti-biofilm activities of ampicillin (AMP), ceftriaxone (CEF), and combination treatments of antibiotics and sulbactam (AMP + SUL and CEF + SUL) were evaluated against antibiotic-sensitive *S*. Typhimurium ATCC 19585 (ST^AS^) and clinically isolated multidrug-resistant (MDR) *S*. Typhimurium CCARM 8009 (ST^MDR^). Compared to the control, the minimum inhibitory concentrations (MICs) of AMP against ST^AS^ and CEF against ST^MDR^ were decreased from 32 to 16 μg/mL and 0.25 to 0.125 μg/mL, respectively, in the presence of SUL. The numbers of ST^MDR^ treated with AMP + SUL and CEF + SUL were effectively reduced by more than 2 logs after 4 h of incubation at 37 °C. The β-lactamase activities of ST^AS^ and ST^MDR^ treated with AMP and CEF were reduced from 3.3 to 2.6 μmol/min/mL and from 8.3 to 3.4 μmol/min/mL, respectively, in the presence of SUL. The biofilm cell numbers of ST^AS^ and ST^MDR^ were reduced at all treatments after 24 h of incubation at 37 °C. The biofilm cell numbers of ST^AS^ and ST^MDR^ were reduced by more than 2 logs in the presence of SUL compared to the AMP and CEF alone. The lowest relative fitness level was 0.6 in ST^AS^ treated with AMP + SUL, while no significant differences in the relative fitness were observed in ST^MDR^. This study suggests that β-lactamase inhibitors (BLIs) could be used for controlling biofilm formation of β-lactamase-producing multidrug-resistant *S*. Typhimurium.

## 1. Introduction

Since the discovery of penicillin, bacteria have continuously developed antibiotic resistance, leading to the antibiotic treatment failure in infectious diseases [1]. *Salmonella* Typhimurium is one of the major foodborne pathogens isolated in humans and animals responsible for severe infections [2,3]. The β-lactam antibiotics, particularly third and fourth generation cephalosporins, have been used to treat infections caused by multidrug resistant (MDR) bacteria [4]. These β-lactam antibiotics that target the bacterial cell wall covalently bind to PBPs and then inhibit the synthesis of peptidoglycan [5]. The mechanisms of antibiotic resistance in bacteria include enzymatic degradation, target site modification, reduced permeability, efflux pump activity, horizontal gene transfer, and mutational resistance [6,7]. Among these mechanisms, the resistance to β-lactams is mainly attributed to the production of β-lactamases. In addition to the enzymatic degradation of antibiotics, the alteration in penicillin-binding proteins (PBPs) are also associated with the β-lactam resistance in bacteria, resulting in a decrease in antibiotic binding affinity to PBPs [8]. This has led to the emergence of β-lactam resistance, which has become a serious threat to public health worldwide [9].

Recently, the use of β-lactamase inhibitors (BLIs), including clavulanic acid (CA), sulbactam (SUL), tazobactam (TB), avibactam (AB), and vaborbactam (VB), has gained attention as an alternative method with which to enhance the antimicrobial activity of β-lactams [10]. The commercial combination drugs of β-lactams and BLIs has been used for treating infectious diseases, for instance, ampicillin + SUL, piperacillin + TB, ceftolozane + TB, ceftazidime + AB, and meropenem + VB against MDR bacteria [11,12]. Particularly, SUL has intrinsic antibacterial activity against Gram-negative bacteria producing class A and C β-lactamases [13]. The combined treatment of BLIs with β-lactam antibiotics can enhance susceptibility to β-lactam antibiotics [14]. In addition, the BLIs can protect β-lactam antibiotics from degradation by β-lactamases. The β-lactam-peptidoglycan binding affinity is enhanced in the absence of β-lactamase, resulting in subsequent inhibition of bacterial biofilm formation. Hence, the inhibition of β-lactamases plays an important role in the control of biofilm formation. However, there is still a challenging question of whether BLIs can contribute to the inhibition of biofilm formation [15]. Therefore, the aim of this study was to assess the effect of β-lactam + BLI combination on the inhibition of biofilm formation in MDR *Salmonella* Typhimurium.

## 2. Results

### 2.1. Antibiotic Susceptibility of Salmonella Typhimurium Treated with Sulbactam 

**Figure 1:**
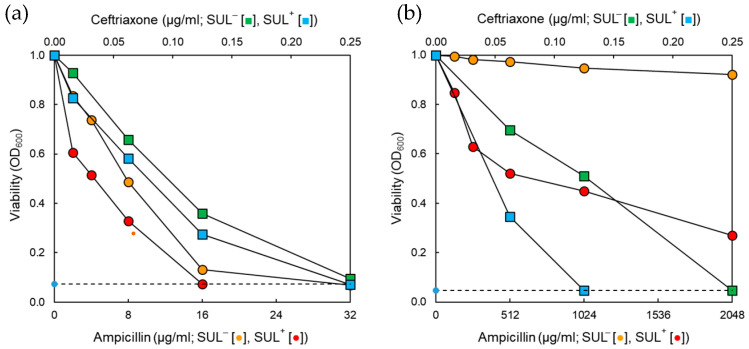
Antibiotic susceptibilities of *Salmonella* Typhimurium ATCC 19585 (ST^AS^); (**a**) and *S*. Typhimurium CCARM 8009 (ST^MDR^); (**b**) treated with ampicillin (AMP; •), ampicillin combined with sulbactam (AMP + SUL; •), ceftriaxone (CEF; ■), and ceftriaxone combined with sulbactam (CEF + SUL; ■) at 37 °C for 18 h.

The antibiotic susceptibilities of ST^AS^ and ST^MDR^ were determined in the absence and presence of sulbactam (SUL) (Figure 1). The susceptibilities of antibiotic-sensitive *S*. Typhimurium ATCC 19585 (ST^AS^) and clinically isolated multidrug-resistant (MDR) *S*. Typhimurium CCARM 8009 (ST^MDR^) to ampicillin (AMP) and ceftriaxone (CEF) were increased in the presence of SUL (Figure 1a,b). The MIC value of AMP against ST^AS^ in the absence of SUL was 32 μg/mL, while that was decreased to 16 μg/mL in the presence of SUL (Figure 1a). The MIC of AMP against ST^MDR^ were not determined, but the viability of ST^MDR^ was significantly decreased in the presence of SUL (Figure 1b). The MIC values of CEF against ST^MDR^ in the absence and presence of SUL were 0.25 and 0.125 μg/mL, respectively. 

### 2.2. Synergistic Effect of Antibiotic and β-Lactamase Inhibitor Combinations on the Growth of Salmonella Typhimurium

**Figure 2:**
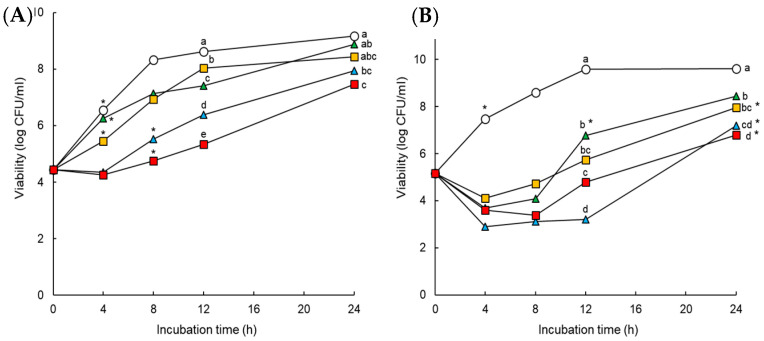
Survival curves of *Salmonella* Typhimurium ATCC 19585(ST^AS^; (**A**) and *S*. Typhimurium CCARM 8009 (ST^MDR^; (**B**) exposed to untreated control (CON; ο), ampicillin (AMP; ▲), ampicillin with sulbactam (AMP + SUL; ▲), ceftriaxone (CEF; ■), and ceftriaxone with sulbactam (CEF + SUL; ■) at 37 °C for 24 h. Different letters (a–e) indicate significant difference among treatments at 12 and 24 h and asterisk (*) denotes significant increase in growth compared to the inoculum at *p* < 0.05.

The antimicrobial activities of AMP and CEF in the absence and presence of SUL against ST^AS^ and ST^MDR^ were evaluated at 37 °C for 24 h (Figure 2). Compared to the control, the growth of ST^AS^ and ST^MDR^ were delayed by all treatments throughout the incubation time (Figure 2A,B). ST^AS^ treated with AMP and CEF showed a significant increase in growth at early incubation time (Figure 2A). However, the cell growth was slowed down when treated with AMP + SUL and CEF + SUL after 4 h of incubation time. The numbers of ST^MDR^ treated with AMP + SUL and CEF + SUL were reduced to less than 4 log CFU/mL at the early stage of incubation time (<6 h), followed by a rapid growth recovery at the late stage of incubation (>7 log CFU/mL) (Figure 2a).

### 2.3. Effect of β-Lactam/β-Lactamase Inhibitor Combinations on the β-Lactamase Activity of Salmonella Typhimurium 

**Figure 3:**
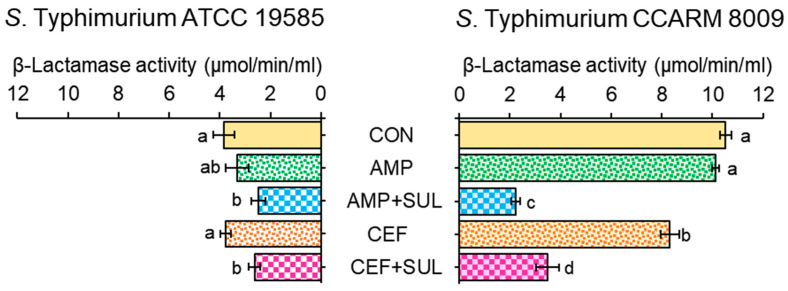
Hydrolyzing activity of β-lactamases produced by *Salmonella* Typhimurium ATCC 19585 (ST^AS^) and *S*. Typhimurium CCARM 8009 (ST^MDR^) exposed to untreated control (CON), ampicillin (AMP), ampicillin with sulbactam (AMP + SUL), ceftriaxone (CEF), and ceftriaxone with sulbactam (CEF + SUL). Means with different letters (a–d) on the bars within ST^AS^ and ST^MDR^ are significantly different at *p* < 0.05.

The membrane-bound β-lactamase activities of ST^AS^ and ST^MDR^ were measured in the absence and presence of SUL (Figure 3). The β-lactamase activities were higher in ST^MDR^ than ST^AS^ at all treatments. No significant difference in β-lactamase activities of ST^AS^ was observed between AMP and CEF treatments, showing 3.3 and 3.7 μmol/min/mL, respectively. The sulbactam effectively inhibited the β-lactamase activities of ST^AS^ which were decreased to 2.3 and 2.6 μmol/min/mL, respectively, at AMP + SUL and CEF + SUL. The highest β-lactamase activity was observed for ST^MDR^ at the control (10.5 μmol/min/mL), followed by AMP (10.1 μmol/min/mL) and CEF (8.3 μmol/min/mL). These β-lactamase activities of ST^MDR^ were significantly decreased to 2.2 μmol/min/mL for AMP + SUL and 3.2 μmol/min/mL for CEF + SUL.

### 2.4. Role of β-Lactamase Inhibitor in Biofilm-Forming Ability and Relative Fitness of Salmonella Typhimurium

**Figure 4:**
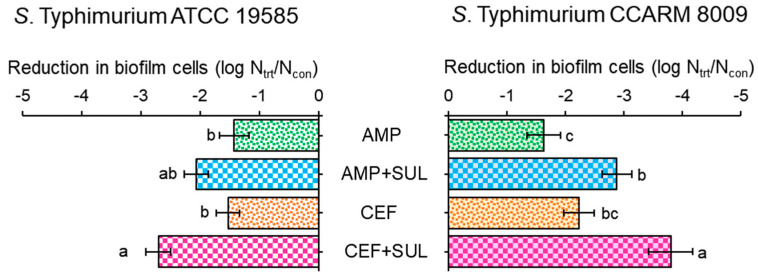
Reduction in *Salmonella* Typhimurium ATCC 19585 (ST^AS^) and *S*. Typhimurium CCARM 8009 (ST^MDR^) treated with 1/2 MICs of ampicillin (AMP) and 1/2 MICs of ceftriaxone (CEF) cultured in the absence (AMP and CEF) and presence (AMP + SUL and CEF + SUL) of sulbactam. Log reduction was estimated as compared to control. Means with different letters (a–c) on the bars are significantly different at *p* < 0.05 within treatments among treatments.

The biofilm-forming abilities of ST^AS^ and ST^MDR^ were evaluated in the absence and presence of SUL (Figure 4). Compared to control, the biofilm cell numbers of ST^MDR^ treated with AMP and AMP + SUL showed 1.6 and 2.9 log reductions, respectively. A noticeable reduction in biofilm cell numbers of ST^MDR^ was observed, showing 2.2 and 3.8 log reductions between CEF and CEF + SUL, respectively. The biofilm cell numbers of ST^AS^ showed 1.4 log reductions at AMP, whereas an increased reduction (2.9 log) was observed for AMP + SUL. Similarly, the biofilm cell numbers of ST^AS^ treated with CEF and CEF + SUL showed 1.5 and 2.7 log reductions, respectively.

**Figure 5:**
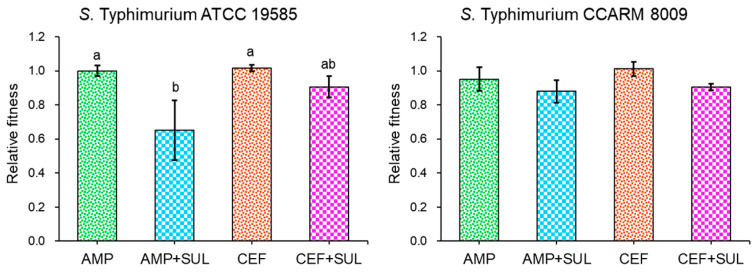
Relative fitness of *Salmonella* Typhimurium ATCC 19585 (ST^AS^) and *S*. Typhimurium CCARM 8009 (ST^MDR^) treated with 1/2 MICs of ampicillin and 1/2 MICs of ceftriaxone cultured in the absence (AMP and CEF) and presence (AMP + SUL and CEF + SUL) of sulbactam. Means with different letters within ST^AS^ (a–b) different at *p* < 0.05.

The relative fitness of resistance was estimated in ST^AS^ and ST^MDR^ cultured in the absence and presence of SUL (Figure 5). ST^AS^ treated with AMP + SUL showed the lowest relative fitness (0.6), whereas no significant difference in relative fitness was observed in ST^MDR^ treated with CEF + SUL. The relative fitness levels of ST^AS^ treated with AMP and AMP + SUL were 1 and 0.6, respectively. Similarly, the relative fitness levels of ST^AS^ treated with CEF and CEF + SUL were 1 and 0.9, respectively. No significant difference in relative fitness levels was found between AMP and AMP + SUL, showing 0.9 and 0.8, and with CEF and CEF + SUL showing 1 and 0.9 against ST^MDR^, respectively.

## 3. Discussion

This study describes the influence of SUL on the inhibition of biofilm formation of ST^AS^ and ST^MDR^. Although the BLIs have been well known as adjuvants that can restore the antimicrobial activity of β-lactams against bacteria [16], there is still a lack of information on the effect of BLIs in association with biofilm formation. *Salmonella* Typhimurium mainly causes gastroenteritis, enteric fever, bacteremia, urinary tract infections, and pneumonia [17]. Therapeutic failure arises due to the inefficacy of conventional antibiotics, causing high morbidity and mortality [18]. β-lactamases are the major resistance determinant for β-lactam antibiotics in *S*. Typhimurium. The inhibition of these β-lactamases can increase the susceptibility of *S*. Typhimurium to penicillins, cephalosporins, and carbapenems. Therefore, BLIs can be used to overcome the β-lactam resistance mechanisms in MDR bacteria.

The susceptibilities of ST^AS^ and ST^MDR^ to CEF were increased in the presence of SUL (Figure 1), indicating that SUL effectively inactivated β-lactamases (TEM, SHV, CTX-M) produced by *S*. Typhimurium [16,19]. This is in agreement with previous studies that the combinations of SUL with β-lactam antibiotics potentiated the antimicrobial activities against Gram-negative bacteria by enhancing antibiotic binding affinity to PBPs [20,21]. SUL irreversibly binds to the active site of β-lactamases, resulting in the enzyme inactivation and an increase in antibiotic stability [14]. Therefore, the increased susceptibility might be attributed to the inactivation of class A β-lactamases expressed in *S*. Typhimurium. AMP did not affect the growth of ST^MDR^ in the presence of SUL. The mutation in PBPs such as PBP3, PBP4, and PBP6 can confer the resistance of *S*. Typhimurium to β-lactam antibiotics by reducing the entry into porin channels [22]. In addition, bacteria can expel toxic substances such as β-lactams through multi-drug efflux pumps, leading to an increased resistance to β-lactam antibiotics [23]. Therefore, the combination of β-lactam + BLIs plays an important role in the control of β-lactam resistance in bacteria. 

The antibiotic activities of AMP and CEF against ST^AS^ and ST^MDR^ were enhanced in the presence of SUL (Figure 2). Compared to the control, the growth of ST^AS^ exposed to AMP or CEF and SUL was constantly inhibited up to 4 h of incubation, followed by a significant recovery after 24 h. The bacterial cell numbers of ST^MDR^ were most effectively reduced up to 12 h of incubation, when exposed to AMP + SUL. The delay in bacterial growth indicates that the degradation of AMP and CEF was negligible when combined with SUL, demonstrating that SUL effectively protects AMP and CEF activity from hydrolysis by β-lactamases. Therefore, the resistance to penicillin and cephalosporins might be due to the production of hydrolytic enzymes in bacteria [13]. In addition, the combination treatments of β-lactams and BLIs effectively restored the antimicrobial activity against antibiotic-resistant, Gram-negative bacteria [16]. Furthermore, the combination of ampicillin with SUL effectively extended the antibacterial spectrum against antibiotic-resistant bacteria [24]. This confirms the low growth rate of ST^MDR^ in the presence of AMP + SUL. The combination treatments of β-lactams and BLIs, including piperacillin-TB, ceftazidime-AB, ampicillin-SUL, amoxicillin-CA, and imipenem-relebactam (RB) are frequently used to treat infections caused by β-lactamase-producing bacteria such as *Escherichia coli*, *Klebsiella pneumoniae*, *Acinetobacter baumannii* [25]. Therefore, BLIs can be used to treat infections caused by MDR bacteria by enhancing the susceptibility of bacteria to β-lactam antibiotics.

The high β-lactamase activity was obtained in ST^MDR^ when compared to ST^AS^ exposed to untreated control, AMP, AMP + SUL, CEF, and CEF + SUL (Figure 3). The significant reduction in β-lactamase activity of ST^MDR^ cells treated with AMP + SUL and CEF + SUL might be attributed to the production of different β-lactamases that was inhibited by SUL [26]. This implies that the resistance to β-lactam antibiotics might be due to the high production of β-lactamases in ST^MDR^, corresponding to the decreased MIC values in the presence of SUL (Figure 1) [27]. The SUL enhanced the activity of β-lactam antibiotics against *A*. *baumannii* [28]. Therefore, the use of BLIs has gained great attention in controlling β-lactamase-producing bacteria. Other than the β-lactamase production, the mutations can result in resistance to β-lactam antibiotics [29]. For example, the mutations of *cpxA* contribute to the resistance to aminoglycosides and β-lactams against *S*. Typhimurium [30]. In addition, exposure to β-lactam antibiotics induces the upregulation of genes which encode the expression of β-lactamase in Gram-negative bacteria [8]. Therefore, the inhibition of β-lactamase activity might be a good strategy to control the antibiotic-resistant bacteria by preventing the hydrolysis of β-lactam antibiotics.

The formation of complex biofilms is a survival strategy of bacteria exposed to stressful conditions such as antibiotic treatment, bile salts, low oxygen, and nutrient depletion [31]. Biofilm cells are firmly imbedded in a complex matrix of extracellular polymeric substances (EPS), which prevents the entry of antibiotics into bacteria [23]. The high biofilm numbers were observed in ST^MDR^ treated with AMP and CEF. This might be due to the enhanced β-lactamase activity in ST^MDR^, suggesting that β-lactamase-mediated hydrolysis of β-lactams can prevent the attack on the peptidoglycan, enabling biofilm formation [32]. A previous study has shown that biofilm forming ability was high in extended-spectrum, β-lactamase-producing bacteria corresponding to the increased resistance to antibiotics, including aminoglycosides, fluoroquinolones, macrolides, and cephalosporins [33]. In addition, sub-inhibitory concentrations of antibiotics can induce biofilm formation in bacteria. This is in agreement with a previous study in which the sub-inhibitory concentrations of antibiotics were found to trigger the release of extracellular DNA, contributing to biofilm formation in bacteria [34]. SUL effectively restored the antibiotic activity of AMP and CEF against ST^AS^ and ST^MDR^ biofilm cells. This confirms that BLIs play a role in the inhibition of biofilm formation in Gram-negative bacteria. A previous study showed that SM23, a derivative of boronic acid, as BLI, could inhibit biofilm formation in *Pseudomonas aeruginosa* by interfering with genes involved in biofilm formation, quorum sensing system, and expression of virulence factors [35]. Furthermore, BLI could downregulate the expression of quorum sensing-associated autoinducers 3-oxo-C_12_-HSL and C_4_-HSL during biofilm formation by lowering the expression of the *lasI/lasR* gene [35]. This implies that competitive binding of BLIs to the LasR receptor inhibits the synthesis of autoinducer molecules, resulting in reduced biofilm formation. Therefore, targeting biofilm and quorum sensing-related genes might be an alternative tool to inhibit biofilm formation. 

The low relative fitness values were observed in ST^AS^ treated with AMP + SUL (Figure 5). This suggests that ST^AS^ treated with AMP + SUL showed a decrease in mutant frequency due to the high fitness cost [36]. The bacterial viability is related to the relative fitness. However, no significant difference in relative fitness was observed for ST^MDR^-treated AMP, CEF, AMP + SUL, and CEF + SUL. This might be due to the high level of antibiotic resistance, leading to bacterial cross-resistance to other antibiotics [37]. The antibiotic resistance in bacteria can be associated with a biological fitness cost [38]. The magnitude of the fitness influences the rate of resistance development and stability [39]. Moreover, the low fitness cost means that bacteria are more likely to adapt when exposed to antibiotics. Eventually, the high relative fitness can reduce the level of the cost of biological fitness [40]. Furthermore, the compensatory mutations improve fitness in genotypes that contain deleterious mutations but have no beneficial effects [41]. The bacterial fitness is associated with the evolution and maintenance of antibiotic resistance. 

## 4. Materials and Methods

### 4.1. Bacterial Strains and Culture Conditions 

Strains of *Salmonella enterica* serovar Typhimurium ATCC 19585 (ST^AS^) and *S.* Typhimurium CCARM 8009 (ST^MDR^) were obtained from American Type Culture Collection (ATCC, Manassas, VA, USA) and Culture Collection of Antibiotic Resistant Microbes (CCARM, Seoul, Korea), respectively. All strains were cultured in trypticase soy broth (TSB; Difco, BD, Sparks, MD, USA) at 37 °C for 20 h. The activated cells were harvested by centrifugation at 6000× *g* for 10 min at 4 °C and then resuspended with phosphate-buffered saline (PBS; pH 7.2) to adjust to 10^8^ CFU/mL.

### 4.2. Antibiotic Susceptibility Assay

The minimum inhibitory concentrations (MICs) of ampicillin (AMP) and ceftriaxone (CEF) against ST^AS^ and ST^MDR^ were determined using a broth microdilution method. Antibiotic stock solutions were prepared to obtain a final concentration of 1024 μg/mL dissolved in water. Each antibiotic stock solution (100 μL) was serially (1:2) diluted with TSB in 96-well microtiter plates and 10^5^ CFU/mL of test strains were inoculated with and without sulbactam (SUL; Sigma Chemicals; St Louis, MO, USA) at 8 μg/mL. The plates were incubated at 37 °C for 18 h to determine MICs that are the lowest concentrations, where no bacterial growth was observed.

### 4.3. β-Lactamase Activity Assay

The β-lactamase activity of ST^AS^ and ST^MDR^ was evaluated using the nitrocefin-hydrolyzing assay with minor modifications [42]. Each strain (10^5^ CFU/mL) was cultured in no antibiotic, 1/2 MIC AMP, 1/2 MIC CEF, AMP + SUL, and CEF + SUL at 37 °C for 20 h. The suspension containing bacteria cells was mixed with 10 μl of 1.5 mM nitrocefin and incubated at 37 °C for 1 h. The absorbance was measured after 1 h of incubation at 512 nm using a microplate reader (BioTek Instruments, Inc., Norwood, MA, USA).

### 4.4. Biofilm-Forming Ability Assay

The ability of ST^AS^ and ST^MDR^ to form biofilm was evaluated in the absence and presence of antibiotics. The bacterial cells (10^5^ CFU/mL) were inoculated in a 96-well microtiter plates containing no antibiotic, 1/2 MIC AMP, 1/2 MIC CEF, AMP + SUL, and CEF + SUL. After 24-h incubation at 37 °C, each microplate well was rinsed with PBS. The adhered cells were harvested using a cell scraper (Thermo Scientific Nunc, Rochester, NY, USA), suspended in PBS, and then serially diluted (1:10) with PBS. Each dilution was plated on TSA using an Autoplate Spiral Plating System (Spiral Biotech, Inc., Norwood, MA, USA). The plates were incubated at 37 °C for 24 h and the attached cells were enumerated using a QCount^®^ Colony Counter (Spiral Biotech, Inc., Norwood, MA, USA).

### 4.5. Estimation of Relative Fitness

The relative fitness of ST^AS^ and ST^MDR^ treated with AMP, CEF, AMP + SUL, and CEF + SUL was determined at 37 °C for 24 h. The relative fitness was estimated as the ratio of the growth (OD_600_) of antibiotic-treated ST^AS^ and ST^MDR^ cells and the untreated cells cultured in antibiotic-free media.

### 4.6. In Vitro Time-Kill Assay

Time-kill assay was performed to determine the antimicrobial activity of AMP, CEF, AMP + SUL, and CEF + SUL against ST^AS^ and ST^MDR^. Approximately 10^5^ CFU/mL of bacterial strains were inoculated with no antibiotic, 1/2 MIC AMP, 1/2 MIC CEF, and combinations of antibiotics and SUL (10 μg/mL). Samples were incubated at 37 °C for 24 h in a shaking incubator at 180 rpm. Viable counts were determined at 0, 4, 8, 12, and 24 h.

### 4.7. Statistical Analysis

Data were analyzed using Statistical Analysis System (SAS). The general linear model (GLM) procedure and Fisher’s least significant difference (LSD) were used to determine significant difference among treatments at *p* < 0.05, *p* < 0.01, and *p* < 0.001. 

## 5. Conclusions

This study describes the effects of the β-lactamase inhibitor on the inhibition of biofilm formation in *S*. Typhimurium. The most significant findings of this study were that: (1) SUL significantly enhanced the susceptibilities of ST^AS^ and ST^MDR^ to AMP and CEF; (2) the β-lactamase activities of ST^AS^ and ST^MDR^ were reduced in the presence of SUL; (3) the biofilm-abilities of ST^AS^ and ST^MDR^ were inhibited when treated with AMP + SUL and CEF + SUL. CEF + SUL showed the highest anti-biofilm activity against ST^AS^ and ST^MDR^. Therefore, β-lactam/β-lactamase inhibitors can effectively inhibit the biofilm formation of ST^AS^ and ST^MDR^. This suggest that BLI-based combinations can be used as an alternative anti-biofilm therapy. 

## Data Availability

Not applicable.
